# Time-Sensitive Networking to Improve the Performance of Distributed Functional Safety Systems Implemented over Wi-Fi

**DOI:** 10.3390/s23187825

**Published:** 2023-09-12

**Authors:** Alberto Morato, Stefano Vitturi, Federico Tramarin, Claudio Zunino, Manuel Cheminod

**Affiliations:** 1 National Research Council of Italy, CNR-IEIIT, 35131 Padova, Italy; alberto.morato@cnr.it; 2 Department of Engineering “Enzo Ferrari”, University of Modena and Reggio Emilia, 41125 Modena, Italy; federico.tramarin@unimore.it; 3 National Research Council of Italy, CNR-IEIIT, 10129 Torino, Italy; claudio.zunino@cnr.it (C.Z.); manuel.cheminod@cnr.it (M.C.)

**Keywords:** functional safety networks, Industry 4.0, Industrial Internet of Things, industrial wireless networks

## Abstract

Industry 4.0 has significantly improved the industrial manufacturing scenario in recent years. The Industrial Internet of Things (IIoT) enables the creation of globally interconnected smart factories, where constituent elements seamlessly exchange information. Industry 5.0 has further complemented these achievements, as it focuses on a human-centric approach where humans become part of this network of things, leading to a robust human–machine interaction. In this distributed, dynamic, and highly interconnected environment, functional safety is essential for adequately protecting people and machinery. The increasing availability of wireless networks makes it possible to implement distributed and flexible functional safety systems. However, such networks are known for introducing unwanted delays that can lead to safety performance degradation due to their inherent uncertainty. In this context, the Time-Sensitive Networking (TSN) standards present an attractive prospect for enhancing and ensuring acceptable behaviors. The research presented in this paper deals with the introduction of TSN to implement functional safety protocols for wireless networks. Among the available solutions, we selected Wi-Fi since it is a widespread network, often considered and deployed for industrial applications. The introduction of a reference functional safety protocol is detailed, along with an analysis of how TSN can enhance its behavior by evaluating relevant performance indexes. The evaluation pertains to a standard case study of an industrial warehouse, tested through practical simulations. The results demonstrate that TSN provides notable advantages, but it requires meticulous coordination with the Wi-Fi MAC layer protocol to guarantee improved performance.

## 1. Introduction

Factory automation has undergone a profound revolution thanks to the introduction of the Industrial Internet of Things (IIoT) concept. In this context, information and communication technologies (ICT) are ever more used to create smart factory ecosystems comprising distributed networks of objects capable of seamlessly interacting. Since the introduction of such technologies, the push for innovation has continued to grow, as witnessed by the industrial revolution, referred to as Industry 4.0 [1], which has been recently followed by Industry 5.0 [2,3].

Basically, Industry 4.0 is a concept introduced about a decade ago which relies on the intelligent networking among machines, controllers, sensors and actuators, and processes in general, to implement digitized production systems [4]. This technology-driven paradigm enhances the manufacturing capabilities, improving production efficiency and flexibility, while ensuring energy efficiency, rational use of resources, and environmental sustainability.

Industry 5.0 is a value-driven paradigm that is designed to complement and enhance the aforementioned features. Its origin can be traced back to pioneering works, such as [5], and has since been adopted by the European Commission [6]. Industry 5.0 focuses on three main concepts, namely, the human-centric approach, sustainability, and resilience [7]. Particularly, the human-centric approach involves the direct participation of humans in the production processes to facilitate robust, profound, and fruitful cooperation with machines. In this respect, a common example involves the interaction between humans and collaborative robots (CoBots).

The innovative context described above poses further requirements for the communication systems deployed in the industrial scenario [8]. Two fundamental issues need to be addressed: (i) the pervasive use of industrial wireless networks and (ii) the implementation of robust and effective distributed functional safety systems. In this regard, as collaboration between individuals and machines is essential, ensuring a high level of protection is crucial for both operators and the surrounding environment. Therefore, functional safety systems are of the utmost importance [9]. Thus, in the Industry 4.0–5.0 scenarios, it is necessary to implement robust, reliable, and safe communication protocols over wireless networks. The benefits of this implementation are manifold. Notably, eliminating cabling results in significant cost-savings and greatly enhances the flexibility and scalability of networks.

In this direction, the IEC 61784–3 International Standard [10] defined some effective protocols such as Fail Safe over EtherCAT (FSoE), ProfiSAFE, and OpenSafety that, however, were primarily conceived to work with wired communication systems. Nevertheless, thanks to the “Black Channel Approach” introduced by IEC 61784–3, they can be implemented on wireless networks as well. Unfortunately, the introduction of wireless systems may negatively impact the behavior of the plants that use the functional safety networks, due to the implicit uncertainty of the communication medium. As an example, impairments like a delay in delivery a packet or, worse, a lost packet that occur during the operation of a functional safety network may trigger the intervention of the safety stop function, with the consequent shutdown of the plant.

In this scenario, however, the opportunities offered by Time-Sensitive Networking (TSN) [11,12] may represent a valuable and significant step forward. TSN is a family of standards originally conceived for Ethernet networks that has recently started to be considered for industrial wireless communication systems as well. It includes protocols for distributed device synchronization, traffic shaping and scheduling, and network redundancy (to mention some) that can be profitably used to dramatically improve communication performance, particularly in terms of timeliness and reliability [13].

To the best of the authors’ knowledge, TSN has never been considered to implement functional safety networks, whether wired or wireless. Based on this consideration, this paper addresses the adoption of TSN for functional safety protocols implemented over wireless networks, specifically Wi-Fi. In light of the current state of the art, and particularly with reference to the contribution described in [14], this paper presents some novel elements, as follows:

- A reference functional safety protocol is introduced to make the analysis more general;
- Various Wi-Fi modulation and coding schemes (MCSs) are used;
- Different channel models are taken into account;
- Two TSN protocols, namely, IEEE 802.1AS and IEEE 802.1Qbv, over Wi-Fi are employed.

The analysis is based on the specific case study of an automated warehouse in which robots and humans cooperate to transfer goods from/to different areas of the plant. It is carried out through numerical simulations and focuses on the behavior of two meaningful performance indexes, namely, the safety function response time (*SFRT*) and the percentage of failed pollings (*PFP*), which will be formally defined in the following. The final goal is to assess whether TSN can bring significant performance improvements, as well as to investigate the most suitable configurations of Wi-Fi and TSN in this challenging field of application.

In detail, the rest of the paper is organized as follows. Section 2 reports some related work. Section 3 introduces the model of functional safety protocol which is used as reference protocol throughout the paper. Section 4 provides the theoretical characterization of both SRT and *PFP*. Section 5 describes the automated warehouse used as case study. Section 6 presents the results of the assessment carried out to evaluate the behaviors of *SFRT* and *PFP*. The simulations refer to a functional safety network that adopts the aforementioned reference protocol over Wi-Fi, with or without the use of TSN. Finally, Section 7 concludes the paper.

## 2. Related Work

Functional safety, in general, has been largely addressed by the scientific literature in the past years. However, the contributions in this context are significantly reduced if we refer to functional safety networks, and in particular to those based on wireless communication systems.

In [15], the authors describe the implementation of a proprietary protocol for real-time Ethernet networks that includes safety and security mechanisms. Although the protocol is not among those standardized by IEC 61784-3 [10], the paper provides significant and interesting results obtained from practical tests carried out on a real system implementation. The relationship between functional safety and dependability for industrial electronics system is dealt with in [16]. In addition to presenting a theoretical survey, the paper introduces two interesting case studies. The paper [17] proposes a modification of the popular WirelessHART protocol to introduce security and safety properties. Notably, the authors carried out a practical implementation of the modified protocol for a commercial distributed control system (the 800xA DCS by ABB) and showed the outcome of some experimental sessions conducted to measure the *SFRT*. WirelessHART is addressed also by [18]. The authors introduce an event–triggered mechanism to transmit safety-related information aimed at limiting the bandwidth requested by the typical functional safety protocols that are based on master–slave techniques. The paper includes an analytical description of *SFRT*, as well as its experimental evaluation carried out for a prototype network. In [19], the authors focus on the description of *SFRT* for safety protocols based on wireless networks. Specifically, they consider the IEEE 802.15.4e standard [20] and propose a theoretical model to determine the *SFRT*. The effectiveness of the model is demonstrated by means of a numerical example. The implementations of two different functional safety protocols on Wi-Fi are proposed in [14,21], respectively. In [21], the authors refer to openSAFETY and introduce an implementation that makes use of UDP and MQTT (Message Queuing Telemetry Transport) in combination with TCP. Then, they present a set of experimental tests to measure the safe response time (*SRT*, which accounts for the *SFRT*). In [14], the authors address another popular functional safety protocol, namely, Fail Safe over EtherCAT (FSoE). After providing the analytical description of *SFRT*, they report the results of an experimental assessment in which, mainly, they evaluate the polling time of the slaves, which contributes to determine the *SFRT*.

The analysis shows that the adoption of wireless networks by functional safety protocols presents an interesting opportunity for current and future industrial systems, even though it poses significant and diverse challenges that the scientific community has only recently started to tackle.

## 3. Reference Functional Safety Protocol

The proposed reference protocol is based on a master/slave technique that resembles those adopted by the protocols of the IEC 61784-3. Basically, a master device cyclically polls a set of slave devices to exchange safety PDUs (SPDUs), which contain safety-related data. In detail, such a polling operation can be either *continuous* or *slotted*. In the continuous polling, the master starts querying the first slave and, upon completion of the polling operation, sequentially moves to the following ones. When the last slave has been polled, the master returns immediately to the first one and starts a new cycle.

In the slotted polling, slots of fixed duration are assigned to master and slaves. The polling cycle is determined by the sequence of the slots. The master starts the cycle by sending the SPDUs to the slaves in its slot. Subsequently, each slave is granted to transmit its SFPU in the slot assigned to it. When the slot of the last slave has expired, a new cycle is started with the slot of the master. Clearly, TSN features are particularly helpful to implement such a technique since they allow (i) strict synchronization of the nodes and (ii) assigning of the time slots to the nodes.

In agreement with IEC 61784-3, the reference functional safety protocol implements some countermeasures against communication errors. They are listed in Table 1 and briefly described in the following. The safety CRC is an additional CRC, with respect to that of the underlying communication system, calculated only on the safety PDU. The PDU numbering is a technique that assigns a sequential number to each safety PDU exchanged between master and slave. It is calculated by the protocol and inserted on a specific field of the safety PDUs. The watchdog is a procedure that allows the checking of whether or not a device is alive. Finally, the slave authentication is a further safety technique that allows the master to constantly know the list of slaves authorized to exchange safety information.

The countermeasures described so far are able to detect some specific communication errors, as listed in Table 2 (the detailed description of the communication errors can be found in the IEC 61784-3 International Standard). When one of such errors occurs, if it is detected by the master, this device issues a safe state transition request to all the slaves and then enters the safe state by itself. Conversely, if the error is detected by a slave, this device sends the request to the master, which in turn dispatches it to all the other slaves and then enters the safe state by itself.

## 4. Performance Indexes

This section deals with the definition of the performance indexes addressed in this paper, namely, *SFRT* and *PFP*.

### 4.1. Safety Function Response Time

*SFRT* was defined by IEC 61784-3 as the “worst case elapsed time following an actuation of a safety sensor connected to a network before the corresponding safe state of its safety actuator(s) is achieved in the presence of errors or failures in the safety function channel”. In practice, *SFRT* is the worst-case time needed by a plant to reach a state in which, typically, actuators are no longer powered as the consequence of a request issued by a safety sensor. Notably, the standard specifies that the *SFRT* definition includes errors or failures in the communication channel. This leads to the equations provided in [10,19].

Although *SFRT* is defined for wired systems, it can be seamlessly extended to safety systems implemented over wireless networks. In this case, the uncertainty of the communication medium may add considerable variability to the aforementioned equations.

As a matter of fact, the definition of *SFRT* does not specify the number of “errors or failures in the safety function channel”. Thus, in some contributions (e.g., [17,18]), the calculation is made considering a single failure, whereas [19] extends the analysis including multiple error sources.

Focusing on the reference functional safety protocol, in case of a single failure, for the continuous polling, the *SFRT* can be expressed as

(1) $$SFRT_{C}=2T_{c}^{max}+\max_{i=1,2,\ldots ,n}\left(TO_{i}-T_{p_{i}}\right)$$

whereas, for the slotted polling, it results

(2) $$SFRT_{S}=2P$$

The meaning of the variables used in (1), (2), and those which will follow is reported in Table 3.

Both (1) and (2) were derived considering that a safety issue detected by a slave needs at most one cycle to be notified to the master, and that another cycle is necessary for the master to send the relevant reactions to the slaves. In this interval of time, an error in the safety function channel reflects in a failed polling of a slave, i.e., in a timeout. Consequently, for the continuous polling case, the duration of one of the two cycles is increased by $\max_{i=1,2,\cdots ,n}\left(TO_{i}-T_{p_{i}}\right)$. In the slotted polling, conversely, the polling time of a slave is constant, even in case of a failure; hence, the cycle time is not increased.

The calculation of *SFRT* can be extended to the case of multifailures in the safety function channel. Indeed, for the continuous polling, the failure of the i-th slave causes an increment of *SFRT* of $TO_{i}-T_{p_{i}}$. The limit situation occurs when all the links between master and slaves are interrupted. In this case, *SFRT* for the continuous polling results

(3) $$SFRT_{max}^{C}=2\sum_{i=1}^{n}TO_{i}$$

whereas *SFRT* does not change for the slotted polling ($SFRT_{max}^{S}=2P$). Notably, in this situation, due to the failures, there is no communication between master and slaves. This is very unlikely to happen for wired networks, since it would imply that all the cables connecting the devices are damaged. Conversely, it can not be excluded a priori for wireless systems. Indeed, for example, the communication could be temporary interrupted due to an in-band interference from different systems. It is worth observing that in this situation the slaves are not updated by the master. Nevertheless, they are forced into a safe state by the watchdog function of the functional safety protocol.

### 4.2. Failed Pollings

Both the polling techniques defined for the reference functional safety protocol may be severely impaired by the occurrence of failed pollings. This kind of problem, with reference to Table 2, is caused by errors like corruption, loss, and delay, and may be effectively detected by the watchdog countermeasure defined by IEC 61784–3. Notably, failed pollings assume particular relevance when wireless networks are adopted, since the implicit uncertainty of the physical medium and the possible interference from other wireless communication systems may severely impact the cyclic exchange of SPDUs between master and slaves. Consequently, it was decided to address the percentage of failed pollings (*PFP*) as a meaningful performance index for the reference functional safety protocol. It is defined as

(4) $$PFP=\frac{N_{fp}}{N_{cyc}}\times 100$$

where $N_{cyc}$ is the total number of cycles executed in a given interval of time, and $N_{fp}$ is the number of failed pollings occurred within that interval of time.

For both the techniques adopted by the reference functional safety protocol (continuous and slotted), timeouts are used to implement the watchdog function and, hence, to detect failed pollings. In detail, for the continuous polling they are detected either by a master which does not receive the answer from a queried slave within a given interval of time, or by a slave which does not receive the expected poll request from the master. For the slotted polling, a master detects a failed polling when a slave does not send the SPDU in the slot assigned to it. Similarly, a slave detects a failure if it does not receive the SPDU from the master.

## 5. Case Study

**Figure 1:**
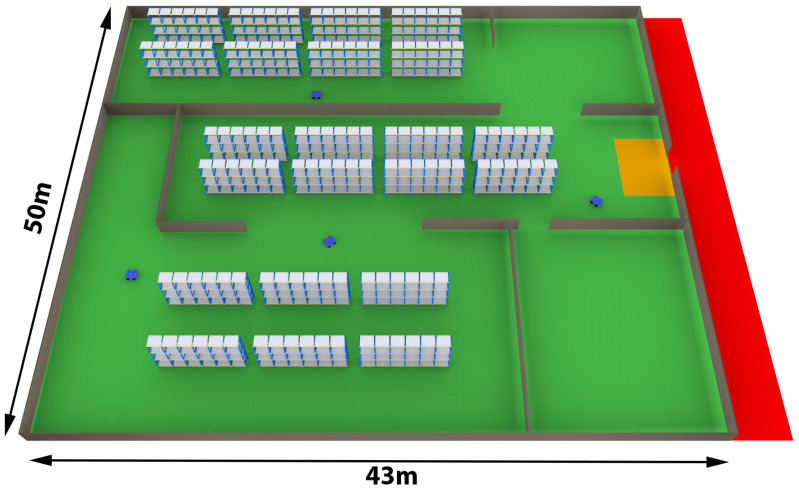
Example of semiautomated warehouse.

The need for functional safety networks based on wireless communication stems from diverse industrial use cases in which mobile devices, for example, robots and AGVs, cooperate among each other and/or with humans in environments where very strict safety requirements are imposed to protect people and machinery. The case study presented in this paper refers to a semiautomated warehouse, schematically represented in Figure 1, that resembles those presented in [22,23], that actually deal with warehouse systems characterized by the contemporaneous presence of mobile devices and humans working in collaboration. The plant includes two distinct workspaces. The first one, highlighted in red, is the loading bay where both the unloading and loading of goods from trucks by human personnel take place manually. The second workspace, highlighted in green, is a fully automated area where mobile robots move goods to/from storage areas for further processing. In this workspace, the access of human personnel is forbidden.

The transfer of goods between the two workspaces takes place through a common area, shown in orange in Figure 1. During operation, human personnel move goods from the red area to the orange one, which are subsequently picked up by robots and brought to the green area. The inverse operation is also possible, with humans operators who pick up goods placed by robots in the orange area and move them to the red one.

Four autonomous mobile robots operate in the automated area. They represent mobile safety slaves connected to a safety master located at the center, 5 m above the ground, of the automated area in order to provide an effective coverage. The connection takes place via a safety network based on the reference protocol introduced in Section 3, and implemented over Wi-Fi. A further safety slave device, in this case static, is represented by the barriers placed at the entrance to the automated area. One of the safety requirements is that when the safety barriers are opened, the Safe Stop 1 (SS1) [24] safety function must be activated in the mobile robots, which consists of a controlled braking procedure followed by the deactivation of the power supply to the electric drives that allow the robots to move. Moreover, there is a keep-out area close to the entrance (the orange rectangle in Figure 1) where robots are are not allowed to move. This area has been designed in such a way that, in case of failure, it is sufficiently large to stop a robot before it reaches the entrance.

The automatic warehouse presented in this section is an appropriate case study to address the topics dealt with by this paper. Indeed, the interaction between humans and robots is explicitly mentioned among the features of Industry 5.0. Thus, the adoption of TSN for the implementation of the functional safety reference protocol, in the context of the warehouse, can be adequately investigated and, in particular, a comparison can be made with the case in which TSN is not used.

## 6. Experimental Assessment

### 6.1. Simulation Setup

The communication among nodes in the environment represented in Figure 1 was simulated using *ns3*. The nodes were set to operate with the IEEE802.11ac standard [25], which actually supports the TSN features, in the 5 GHz band.

Every node is equipped with a single antenna and uses a fixed modulation and coding scheme (MCS), ranging from MCS0 to MCS7, thus allowing a single spatial stream both in Tx and Rx. For the propagation loss model, we considered the TGn channel models [26]. This standard proposes a set of 6 profiles, referred to as A to F. In this model, as described by Equation (5), the loss *L* at the distance *d* is given by a two-slope model where the first part is the free space loss $L_{FS}$ up to a breakpoint distance $d_{BP}$ and slope of 3.5 after the $d_{BP}$, where $d_{BP}$ is defined according to the specific profile.

(5) $$L\left(d\right)=L_{FS}+\left\{\begin{array}{cc} 0, & d<d_{BP}\\ 35\;\cdot \;log(d/d_{BP}) & d\geq d_{BP} \end{array}\right.$$

In the simulation setup, we considered both the TGn model D ($d_{BP}=10$ m) and TGn model E ($d_{BP}=20$ m), since, according to their specifications, they appear to be the most suitable to describe the application environment. Indeed, the TGn model D is typically used in the design and optimization of indoor wireless networks, whereas TGn model E refers to wireless networks that cover both indoor and outdoor environments [26].

In agreement with the description of the reference safety protocol provided in Section 3, we considered the two polling techniques, namely, continuous and slotted. The continuous polling was implemented by using request and response frames issued, respectively, by the master and the slaves. The slotted polling was implemented by exploiting the TSN features defined by IEEE 802.1AS and IEEE 802.1Qbv [27,28], respectively. IEEE 802.1AS is a standard that allows us to achieve time synchronization among distributed nodes, whereas the IEEE 802.1Qbv standard defines a mechanism for the reservation of bandwidth for time-sensitive traffic. It is based on the concept of gates, which are time intervals during which only a specific class of traffic is allowed to be transmitted. With IEEE 802.1Qbv, time-sensitive devices implement their own schedules according to the network requirements, so that data can be timely delivered without collisions with other transmissions.

**Figure 2:**
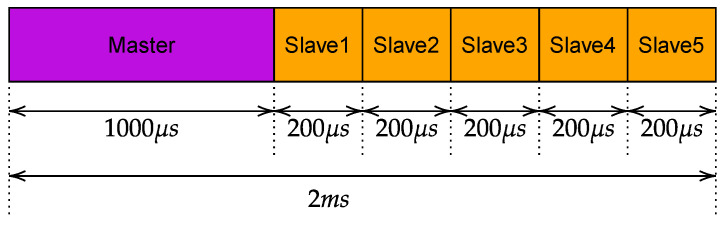
IEEE 802.1Qbv gates schedule.

The duration of the slots was determined considering two different constraints. On the one hand, the duration has to be as small as possible to achieve very low safety response times. On the other hand, the slots have to be big enough to ensure the transmission of the frames. This led to the schedule shown in Figure 2. In practice, the time necessary to safely transmit an SPDU was set to 200 μs. This value was calculated considering the actual time necessary to transmit the SPDU (that carries 8 bytes of safety data and is encapsulated in a User Datagram Protocol (UDP) PDU) over Wi-Fi, and adding a further security margin. Consequently, the master has a slot of duration 2 ms, whereas each slave has 200 μs. The cycle starts with the master slot, in which the SPDUs are sequentially transmitted to the slaves, followed by the slots in which each slave transmits its own SPDU, for a total cycle duration of 2 ms. The watchdog timer was set to 10 ms for all the simulations [29,30].

During operation, the transition of the plant to a safe state can be due to two different causes:

(i) Activation of a safety function by any of the nodes that detects a fault (e.g., a problem of one of the robots);
(ii) Detection of any of the errors reported in Table 2.

Both such causes were considered in the proposed experimental analysis. The *SFRT* was then measured as the time elapsed between the detection of either a fault in the node or the communication error and the achievement of the safe state by all nodes.

In total, 32 different scenarios were analyzed, since for each MCS the behavior of the two polling techniques were simulated for the two channel models.

For every scenario, 30 simulations were performed, where the initial positions of the AGVs, as well as their trajectories, were set randomly.

For every simulation, we collected data about the round trip time and cycle time of SPDUs, *SFRT*, and the cause of transition to the safe state. A recap of meaningful simulation parameters is reported in Table 4.

### 6.2. Behavior of the Two Polling Techniques

**Figure 3:**
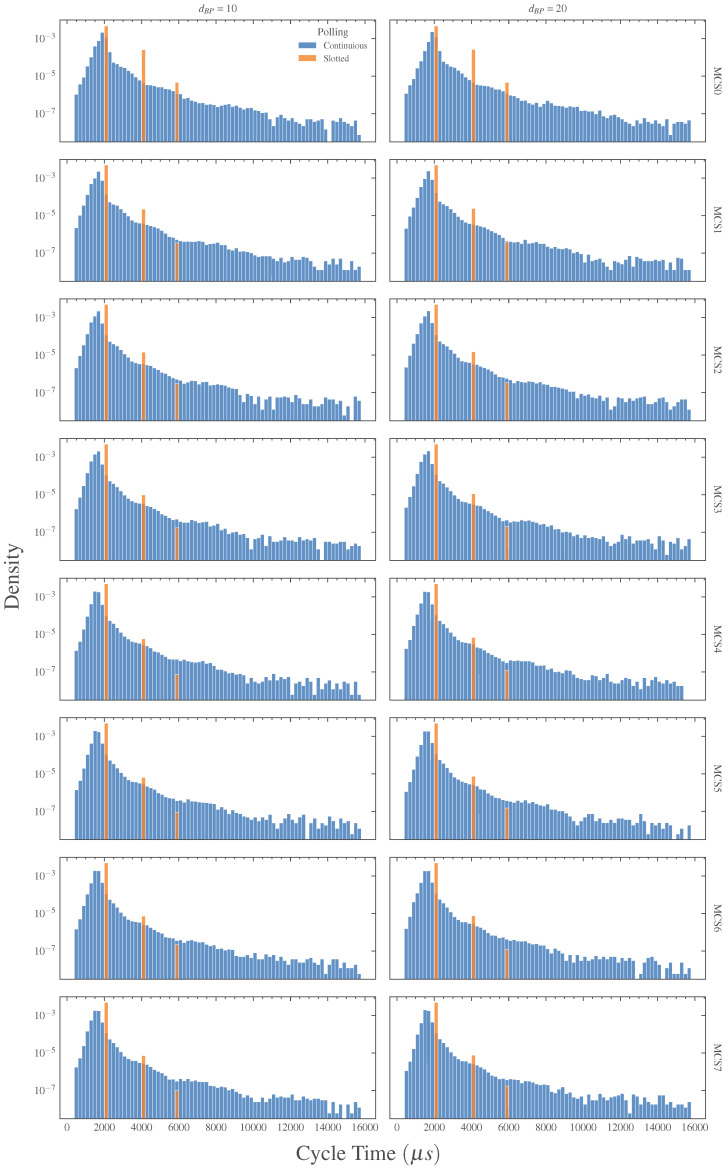
Cycle time densities.

Before addressing the performance of the functional safety protocol, the behavior of the two polling techniques was assessed. In this direction, we investigated the cycle time, $T_{c}$, of the network, defined as the time that elapses between the transmission of two subsequent SPDUs from the master to the same slave. Results are provided in both Figure 3 and Table 5 that report, respectively, the probability density and the statistics of $T_{c}$. As a first comment, the cycle time is not significantly influenced by the selected channel model. This result is not surprising, since the two models are similar and the effects of channel attenuation tend to be more noticeable away from the breakpoint distance, i.e., when the distance between master and slave is significantly greater than $d_{BP}$. Focusing on the actual behavior of $T_{c}$, it may be noticed that, as expected, the continuous technique is characterized by a considerable randomness caused by the lack of an ordered access to the transmission channel. Indeed, with this technique, the master polls the slave without a precise timing and such an operation is heavily influenced by packet losses, retransmissions, random backoff times, etc. Conversely, with the slotted polling, all transmissions are precisely scheduled and the aforementioned problems are mitigated, with the evident benefits on the behavior of $T_{c}$. However, as can be seen, there is still a non-negligible variability. Particularly, the probability density in Figure 3 evidences three bars at 2, 4, and 6 ms, reflecting the fact that some devices (either master or slaves) do not conclude their transmissions in the assigned slot, but require one or two additional slots.

**Figure 4:**
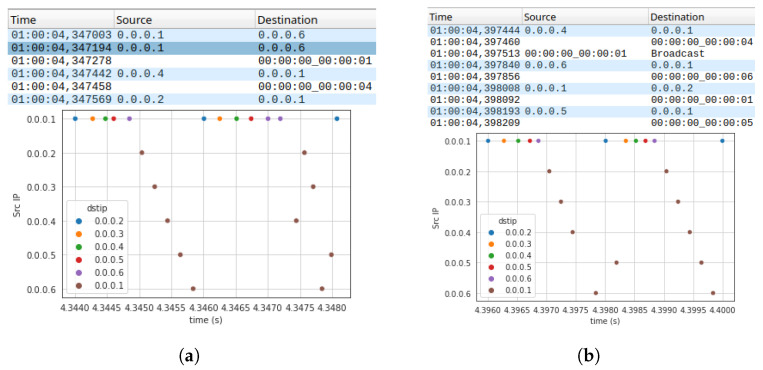
Scheduling problems caused by the MAC layer. Panel (**a**) shows the retransmission of a frame due to the missing ACK; (**b**) shows the presence of a beacon frame that causes the delay of the transmission of a frame. (**a**) The missing ACK of the frame at 4.347003 s causes the retransmission of the frame after a random backoff at 4.347194 s. The transmission overlaps with the window reserved to the device with IP address 0.0.0.2 which is delayed, and in turn compromises the scheduling of the other devices. (**b**) The presence of the beacon frame at 4.397513 s causes the delay of the frame from the device with IP address 0.0.0.5, which performs the transmission in the window reserved for the master.

This is due to the fact that the time-aware scheduler (TAS) of IEEE 802.1Qbv, in some cases, is not able to guarantee the collision-free transmission of SPDUs. Indeed, when a frame is delivered to the MAC layer, the TAS loses control over it. Thus, if the MAC layer delays the transmission of that frame for some reason (e.g., the presence of other transmissions or a random backoff), such a transmission may actually take place in the window reserved by the TAS to another device, compromising the schedule and likely causing additional delays and collisions. Figure 4 shows two possible cases of delays that affect the transmission of SPDUs. In Figure 4a, the missing of an ACK frame causes the retransmission after a random backoff time, whereas in Figure 4b, the window assigned to an SPDU is occupied by the (unexpected) transmission of a beacon frame. A straightforward way to mitigate such a problem is to increase the TAS window duration (i.e., the duration of the slot assigned to safety devices), so that the transmission of SPDUs may take place in the assigned slot, even in the presence of unexpected delays or other inconveniences. Clearly, this solution increases the cycle time and, consequently, reduces the timeliness of the slotted technique. A more preferable solution is to neutralize the causes of delays whenever possible, for example, disabling the sending of beacon frames and preventing Wi-Fi cards from entering idle states such as power save, which has the effect of queuing frame transmission at the MAC layer.

### 6.3. SFRT

**Figure 5:**
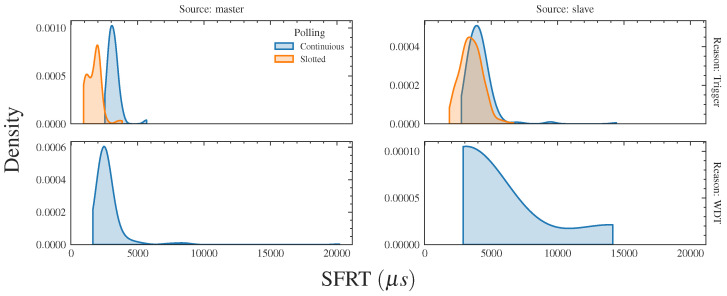
*SFRT* densities.

The empirical probability density function (EPDF) of the *SFRT* is shown in Figure 5, while its statistics are reported in Table 6. In both cases, to improve readability, the presented results are cumulative for all the considered MCSs.

The maximum expected values were calculated according to Equations (2) and (3). We set the cycle time to 2 ms (the watchdog, as mentioned, was 20 ms), resulting in an expected maximum *SFRT* of 200 ms for continuous polling and 4 ms for slotted polling. We separately analyzed the cases in which the safe state transition request is originated either by the master or by a slave. Also, we considered two possible causes of safe state transitions, namely, a trigger issued by any of the devices (typically an emergency request) and the detection of a failed polling by the watchdog countermeasure. As can be seen in Figure 5, when the transition is originated by the master, the safety function response time is lower in comparison with the case in which the transition is originated by a slave. This is understandable since, if the transition is started by the master, ideally, only one network cycle is necessary to activate the safety function on all the slaves. On the other hand, if the transition is originated by a slave, the master has to receive the trigger and then send the transition request to all the other slaves. This requires more than one network cycle.

The analysis of the results shows that the slotted technique (that relies on TSN) performs better than the continuous one, since it allows us to obtain faster *SFRT* values. However, it is interesting to note in Table 6 that, for the slotted technique with the transition to the safe state triggered by a slave, the maximum *SFRT* value overcomes the theoretically calculated one. This is a consequence of the problem evidenced in Section 6.2, where it was observed that in some cases the transmission of SPDUs was delayed with respect to the slots in which it was expected to take place. Clearly, this aspect represents an issue, since ensuring that the safety function response time can be calculated a priori is crucial for a functional safety network. Finally, it has to be mentioned that the missed values in the last row of Table 6 reflect the fact that, for the slotted technique, the slaves did not detect any failed polling.

### 6.4. Percentage of Failed Pollings

The statistics of the percentage of failed pollings (PCP) are reported in Table 7. The presented results are relevant to the whole set of simulations that were executed.

As can be seen, the benefits derived by the adoption of the slotted polling, supported by the TSN features, are evident. Indeed, with the slotted technique, there is a dramatic reduction in the failed pollings. This is an important achievement, since an event of this type causes the transition to the safe state that, as observed in the Introduction, typically implies the shutdown of the plant.

**Figure 6:**
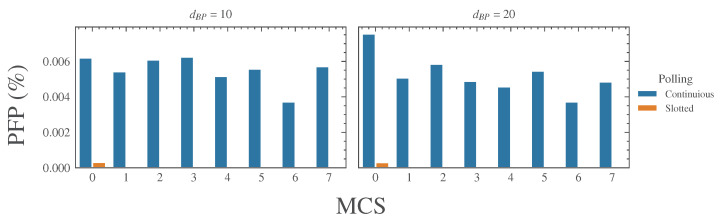
Comparison of failed pollings occurrence.

A further analysis was carried out in order to investigate the possible impact of both the channel loss model and the different MCSs on PCP. Results are presented in Figure 6. It can be observed that *PFP* has a slightly better behavior for the IEEE TGn E loss model. Also, for both channel models, MCS0 performs worse than the other modulation and coding schemes. This is likely due to the long polling times (that are a consequence of the low bit rate) that, in case of additional communication delays, may exceed the timeout.

Generally speaking, as can be seen in Figure 6, the number of failed pollings seems to not have any particular correlation with the MCS and the loss model.

### 6.5. Simulations with a Longer Cycle Time

A second session of simulations was carried out in which a longer duration of the slots was adopted for the slotted technique even if, clearly, this resulted in a longer cycle time. In detail, we assigned a transmission window of 1 ms to each slave, resulting in a cycle time of 10 ms (with the slot of the master set to 5 ms). These simulations were carried out to assess whether increasing the duration of the slots is an effective solution to ensure that devices transmit their SPDUs in the assigned slots without additional delays.

Since in the previous experiments no significant differences were observed in the results obtained for the two propagation-loss models, in this new simulation, only the IEEE TGn D model was used.

The obtained results confirmed the effectiveness of the choice. Actually, for the slotted technique, all the SPDUs were transmitted in the assigned slots; hence, no failed pollings were observed. This was obtained, clearly, at the expense of longer *SFRT* values, as shown in Table 8. For the continuous technique, as expected, no significant performance changes were observed. Indeed, both *SFRT* and *PFP* values were substantially the same as the previous experiment.

### 6.6. Simulations with Best Effort Traffic

**Figure 7:**
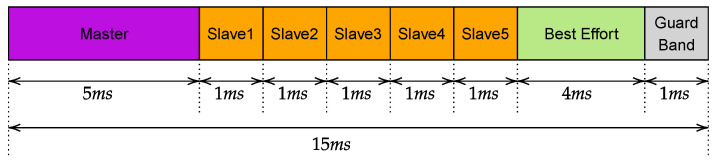
IEEE 802.1Qbv gates schedule with BE traffic.

A final set of simulations was carried out, introducing another type of traffic, namely, best effort (BE), in addition to that relevant to the safety protocol. As in the previous section, we conducted simulations based solely on the IEEE TGn D standard. The BE traffic comprises five TCP streams at a constant rate of 5 Mbps each, simulating video streams transmitted by the slaves to the master during each cycle. Clearly, for the continuous polling technique, since the access to the physical medium is completely unregulated, the safety traffic will result as mixed up with the BE one. Conversely, the slotted technique allows us to add a further window reserved for the BE traffic that, in such a way, becomes separated by the safety one. Figure 7 illustrates the new traffic schedule. As can be seen, in addition to the BE window a new one was added, namely, guard band, that realizes a separation between the two types of traffic, with the aim of ensuring the BE traffic will not influence the safety one. In these experiments, we set the watchdog timeout to 30 ms.

Table 9 presents the statistics of *SFRT*.

**Figure 8:**
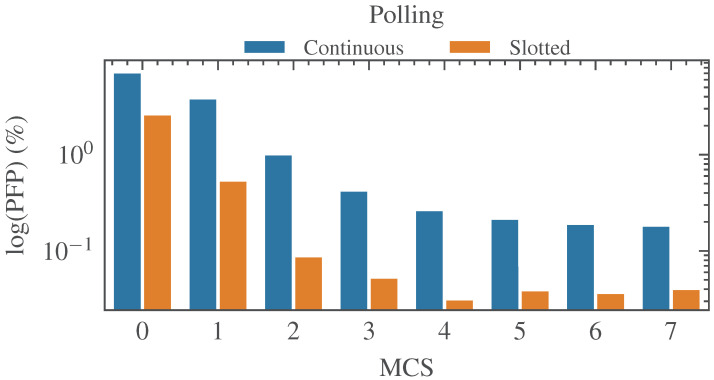
Comparison of failed polling occurrence with BE traffic.

As expected, when using continuous polling, *SFRT* shows a significant degradation compared to the case without BE traffic. Both the standard deviation and the maximum values are considerably higher and, in most cases, are well beyond the maximum expected upper bound. However, also the slotted polling shows a degradation, as is evidenced by the behavior of the *PFP* reported, respectively, in Table 10 and Figure 8.

In actuality, the *PFP* is considerably increased in comparison with the previous cases. The analysis we carried out showed that the problem is caused by the traffic congestion in the BE window. Thus, similarly to the phenomenon described in Section 6.2, packets delivered to the MAC layer to be transmitted in the BE window were moved ahead, overlapping the slots assigned to the safety traffic. An in-depth analysis of the experimental outcomes revealed that the problem is more evident for low MCSs. This is understandable, since the use of low MCSs reflects in longer polling times that are, clearly, more prone to exceeding the timeout and, hence, to causing a polling failure.

Obviously, increasing the size of the guard band windows (and possibly that of all the other windows as well) could represent an immediate solution to the problem; however, this solution has the evident drawback of reducing performance and efficiency. Indeed, as shown in Section 6.5, this causes a worsened behavior of the *SFRT*. Also, the efficiency of the protocol is lowered because it is likely that windows might remain unused for long times.

## 7. Conclusions

In this paper, we conducted an in-depth analysis regarding the possibility of adopting the Time-Sensitive Networking (TSN) family of standards for functional safety networks implemented over wireless systems. The assessment started with the definition a functional safety reference protocol and was followed by an extensive campaign of simulations aimed at investigating the behavior of some meaningful performance indexes. The outcomes demonstrated the indubitable benefits that can be achieved with the adoption of TSN, even if, due to the unavoidable limitations of an analysis based on simulations, some more in-depth studies are necessary to achieve a completely satisfactory assessment.

A very important aspect that emerged from the simulation campaign is that TSN features need to be adequately harmonized with the behavior of the underlying MAC layer to obtain effective results. Indeed, it was shown that frames delivered by TSN to the MAC layer may be actually transmitted with unexpected delays, due to the occurrence of retransmissions of formerly queued frames, random backoff times, and beacons. Such delays have a negative impact on the behavior of the functional safety protocols since both the considered performance indexes (*SFRT* and *PFP*) are worsened. Thus, it is of prominent importance to ensure that frames delivered by TSN to the MAC layer are transmitted while maintaining the specified timing.

Further future activities are expected to extensively address the aforementioned issues. It is envisaged that they will refer to real prototype systems, as well as to simulated environments and theoretical analyses. The implementation of the functional safety reference protocol on real devices is expected to be straightforward, provided that the TSN stack is available for such devices. To this regard, in [31], some preliminary results are provided that report on the implementation of the TSN IEEE 802.1 AS protocol on Intel NUCs (Next Unit Computers). Experiments on real systems will be useful, in particular, to take into account and evaluate additional delays and latency introduced by real components that cannot be determined in other ways. Simulations and theoretical analyses will allow researchers to address more complex systems that typically cannot implemented in practice. Also, the results obtained on the real prototypes can be used to better tune simulation and theoretical models so that they can provide more realistic results.

## Figures and Tables

**Table 1:** Countermeasures implemented by the functional safety reference protocol.

Countermeasure	Description
**Safety cyclic redundancy check (CRC)**	Additional safety-related CRC
**PDU numbering**	Numbering of safety PDUs exchanged between master and slaves
**Watchdog**	Time-out associated with each device
**Slave authentication**	Unique address assigned to each slave and stored by the master

**Table 2:** Error detection by the functional safety reference protocol.

Error	Countermeasures
**Corruption**	CRC and watchdog
**Repetition**	PDU numbering
**Incorrect sequence**	PDU numbering
**Loss**	PDU numbering and watchdog
**Delay**	Watchdog
**Insertion**	Slave authentication
**Masquerade**	CRC and slave authentication
**Addressing**	Slave authentication

**Table 3:** Variables for *SFRT* calculation.

Variable	Meaning
** $T_{p_{i}}$ **	Polling time of Slave *i*
** $T_{p_{i}}^{max}$ **	Maximum polling time of Slave *i*
** $TO_{i}$ **	Timeout Slave *i*
** $T_{c}$ **	Cycle time of the network (continuous polling)
** $T_{c}^{max}$ **	Maximum cycle time of the network
** $T_{sl_{i}}$ **	Slot time of Slave *i*
***P***	Period of the network (slotted polling)

**Table 4:** Simulation parameters.

Parameter	Value
Environment area	50 m × 43 m
AP transmission power	16.0206 dBm
STA transmission power	16.0206 dBm
Number of antennas	1
Spatial streams	1
MCS	0 to 7
Frequency	5 GHz
Number of APs	1 (Master)
Number of STAs	5 (Slaves)
Traffic type	UDP
Propagation loss model	IEEE TGn D and IEEE TGn E
Propagation delay model	Constant speed delay model

**Table 5:** Cycle time statistics.

TSN	$d_{\mathrm{BP}}$	MCS	Mean (μs)	Std (μs)	Min (μs)	Max (μs)
False	10	0	1934.76	468.14	389.00	15,749.00
		1	1680.99	441.00	386.00	15,774.00
		2	1650.95	424.41	387.00	15,781.00
		3	1629.19	400.15	388.00	15,689.00
		4	1629.31	374.46	390.00	15,776.00
		5	1634.01	375.94	400.00	15,764.00
		6	1630.92	371.12	390.00	15,647.00
		7	1618.29	377.75	390.00	15,746.00
	20	0	1967.58	462.97	389.00	15,736.00
		1	1706.94	426.94	386.00	15,711.00
		2	1656.86	429.96	387.00	15,738.00
		3	1635.75	408.02	388.00	15,734.00
		4	1624.73	381.28	390.00	15,367.00
		5	1638.84	362.99	390.00	15,716.00
		6	1626.05	368.42	390.00	15,656.00
		7	1639.32	370.15	399.00	15,695.00
True	10	0	2103.99	452.23	2000.00	6000.00
		1	2009.13	136.94	2000.00	6000.00
		2	2005.95	111.07	2000.00	6000.00
		3	2003.98	90.71	2000.00	6000.00
		4	2002.36	69.50	2000.00	6000.00
		5	2002.66	73.88	2000.00	6000.00
		6	2003.07	80.44	2000.00	6000.00
		7	2002.94	77.65	2000.00	6000.00
	20	0	2108.28	460.59	2000.00	6000.00
		1	2009.87	142.35	2000.00	6000.00
		2	2006.34	114.78	2000.00	6000.00
		3	2004.58	97.29	2000.00	6000.00
		4	2002.80	76.09	2000.00	6000.00
		5	2003.14	80.65	2000.00	6000.00
		6	2003.12	80.11	2000.00	6000.00
		7	2003.15	81.01	2000.00	6000.00

**Table 6:** *SFRT* statistics.

Polling	Reason	Source	Mean (μs)	Std (μs)	Min (μs)	Max (μs)	Expected Maximum (μs)
Continuous	Trigger	Master	3170.77	473.63	2529.00	5670.00	200,000.00
		Slave	4104.05	1134.60	2753.00	14,424.00	200,000.00
	WDT	Master	2808.30	1528.69	1619.00	20,197.00	200,000.00
		Slave	4941.50	4512.01	2878.00	14,143.00	200,000.00
Slotted	Trigger	Master	1700.57	545.05	922.00	3832.00	4000.00
		Slave	3488.00	828.13	1845.00	6677.00	4000.00
	WDT	Master	2015.00	–	2015.00	2015.00	4000.00
		Slave	–	–	–	–	–

**Table 7:** Failed polling statistics.

Polling	Failed Pollings	Total Cycles	Total *PFP* (%)
Continuous	670	12,522,453	0.005350
Slotted	4	10,569,265	0.000038

**Table 8:** *SFRT* statistics for the scenario with 10 ms cycle time.

Polling	Reason	Source	Mean (μs)	Std (μs)	Min (μs)	Max (μs)	Expected Maximum (μs)
Continuous	Trigger	Master	3147.93	750.23	2585.00	7685.00	200,000.00
		Slave	4129.08	1134.98	2342.00	15,408.00	200,000.00
	WDT	Master	2705.95	1002.93	1812.00	11,806.00	200,000.00
		Slave	3449.00	554.37	3057.00	3841.00	200,000.00
Slotted	Trigger	Master	7871.21	1890.23	4129.00	9863.00	20,000.00
		Slave	15,524.01	2711.75	9020.00	19,990.00	20,000.00
	WDT	Master	–	–	–	–	–
		Slave	–	–	–	–	–

**Table 9:** *SFRT* statistics for the scenario with BE traffic.

Polling	Reason	Source	Mean (μs)	Std (μs)	Min (μs)	Max (μs)	Expected Maximum (μs)
Continuous	Trigger	Master	–	–	–	–	–
		Slave	–	–	–	–	–
	WDT	Master	45,948.13	155,899.95	4196.00	1,072,153.00	300,000.00
		Slave	38,611.43	71,221.21	7597.00	580,603.00	300,000.00
Slotted	Trigger	Master	11,850.79	3830.29	5183.00	17,949.00	30,000.00
		Slave	24,534.18	4592.11	14,690.00	33,752.00	30,000.00
	WDT	Master	76,212.50	221,582.99	4047.00	933,050.00	30,000.00
		Slave	46,704.63	127,422.17	14,188.00	900,138.00	30,000.00

**Table 10:** Failed polling statistics with BE traffic.

Polling	Failed Pollings	Total Cycles	Total *PFP* (%)
Continuous	17,629	1,506,478	1.170213
Slotted	1753	613,417	0.285776

## Data Availability

Not applicable.
